# Technical Concepts for the Investigation of Spatial Effects in Spiral-Wound Microfiltration Membranes

**DOI:** 10.3390/membranes9070080

**Published:** 2019-07-04

**Authors:** Martin Hartinger, Hans-Jürgen Heidebrecht, Simon Schiffer, Joseph Dumpler, Ulrich Kulozik

**Affiliations:** 1 Chair of Food and Bioprocess Engineering, Technical University of Munich, 85354 Freising, Germany; 2 Department of Food Science, Cornell University, Ithaca, NY 14853-5701, USA

**Keywords:** length dependency, prototype module, spiral-wound membrane, flat sheet test cell, microfiltration, skim milk, fractionation

## Abstract

Existing works on the influence of spatial effects on flux and permeation of proteins in microfiltration (MF) have focused on ceramic membranes. There is little information on spiral-wound membranes (SWMs). Since the inner core of a SWM is practically inaccessible by non-destructive techniques, three different prototypes were constructed in this study to optimize suitability for the investigation of spatial effects on filtration performance. To measure the pressure drop, shortened SWMs 0.25, 0.50, and 0.75 times the length of a standard industrial SWM (0.96 m) were designed. Second, a sectioned membrane (0.96 m) with separated compartments on the permeate side was constructed to analyze spatial effects on flux and protein permeation along the flow path of a SWM. Three different features characterized this sectioned module: sectioned permeate pockets, a sectioned permeate collection tube, and sectioned permeate drain and measurement systems. Crossflow filtration experiments showed that these modifications did not alter the filtration performance compared to an unmodified control SWM. Thus, it can be applied to assess spatially-resolved filtration performance in SWMs. The third prototype designed was a test cell with accessible flat sheet membranes and spacer material, as in SWMs. The flow path in this test cell was designed to match the characteristics of the channels between the membrane sheets in a standard SWM as closely as possible. The flow path length and the combination of membrane material and spacer architecture were the same as in the control SWM. This test cell was designed to assess the effects of length and processing conditions on the formation of a deposit layer. The combined results of these test modules can yield new insights into the spatial distribution of flux, permeation of target components, and deposit formation.

## 1. Introduction

Milk protein fractionation by means of microfiltration is still an emerging field in the dairy industry. Up to now, ceramic membranes have primarily been used for this application, which have been studied excessively [1,2,3,4,5,6,7,8,9,10]. Polymeric spiral-wound membranes (SWM) provide a possible alternative to ceramic membranes [11,12,13] because of their higher specific membrane area per module and lower filtration operating costs [14]. Filtration performance of both membrane types is affected and limited by deposit layer formation [15,16,17]. The intensity of the deposit layer formation depends on, among other factors, the process variables transmembrane pressure and crossflow velocity.

In crossflow applications, friction losses result in an unavoidable retentate pressure drop along the membrane [18], which leads to a longitudinal reduction of the transmembrane pressure (Δp_TM_). Since the mean Δp_TM_ for microfiltration applications is low in general (0.3–3.0 bar for this application), retentate pressure drop has a strong impact on the deposit layer formation and filtration performance along the membrane. It is known that fouling is more pronounced at higher Δp_TM_ values [17], as well as at a lower crossflow velocity [19]. This means that the higher Δp_TM_ at the membrane inlet causes a more intense deposit layer formation compared to the outlet [20]. These effects have been studied in detail for tubular membranes and flat sheet membranes by Riesmeier [21], and for ceramic membranes by Piry et al. [4,8]. To be able to study the length effects in ceramic membranes, Piry et al. [4] divided a membrane into four sections in axial direction and studied microfiltration-based fractionation of milk proteins. They observed a flux decline along the module, whereas the permeation of the smaller protein fraction increased. The authors concluded that the spatially dependent Δp_TM_ directly affects deposit layer formation by the retained larger proteins (casein micelles) in ceramic tubular membranes, and thus membrane performance. Based on results regarding length-dependent filtration performance in tubular ceramic membranes, different concepts for better control of ceramic membranes were developed. One concept is the uniform transmembrane pressure (UTP) principle, as studied by Kersten [22]. Membranes with a gradient in membrane resistance, as used by Heidebrecht et al. [10], are another possibility. Both systems provide an improved mass flow into the permeate, due to a more homogeneous filtration behavior along the membrane.

Interpreting the observations on ceramic membranes, it seems logical that spatial effects also influence filtration performance in SWMs, but this has not been investigated yet. Reasons for this are most probably the difficulty of measuring spatial effects in SWMs, as the geometry includes complex and narrow spacers which influence the feed flow [23,24,25], and the irregularly curved flow path [26,27]. A direct transfer of the observations from ceramic tubular membranes to SWMs is therefore hardly possible. To assess spatial effects in SWMs, so far, computational fluid dynamics (CFD), test cells, and direct observations have been used, which are helpful tools, but possess certain limitations.

CFD has predominantly been applied to gain more insights into spatial effects in SWMs for reverse osmosis (RO) and nanofiltration (NF) applications, and not for microfiltration [26,28,29,30,31]. The drawback of CFD simulations of flow patterns in SWMs is that major assumptions and simplifications have to be made [28] due to the complex three-dimensional distribution of feed flow velocities, deposit layer formation, and filtration fluid characteristics. Thus, results are subject to uncertainty and require experimental confirmation. For example, Koutsou and Karabelas [32] assumed that the membrane is impermeable, thus, concentration effects were neglected. They proved that simulations can satisfactorily approximate flow patterns. However, this technique cannot be used to assess deposit layer formation and protein permeation. Furthermore, dead zones in SWMs [33] and fouling in the feed channel, inevitable in skim milk microfiltration, lead to an inhomogeneous flow distribution. Schwinge et al. [24] stated that these disparities are difficult to model and represent a major restriction of simulations. Although continuous improvements enhance the accuracy of simulations, assumptions are indispensable. Thus, the accuracy of results is still unsatisfactory, reducing the value of simulations to predict the performance of SWMs, especially for microfiltration applications and low transmembrane pressures without experimental validation.

Another approach is to derive the filtration performance of SWMs from observations in flat sheet test cells [34,35,36,37]. Mo and Ng [35] created a test channel (1.00 m × 0.03 m) to measure protein permeation, flux, and pressure drop along the feed channel in a reverse osmosis application. However, an experimental comparison to the filtration performance of a SWM was not carried out. Siebdrath et al. [37] developed a similar filtration test cell (0.91 m × 0.04 m) for reverse osmosis as well. Although it was indicated that the hydrodynamics are comparable to a SWM, the authors also did not prove that the filtration performance is similar to a real SWM. Due to the small channels, differing boundary conditions occur in the flow channel of a SWM and a test cell. Therefore, a direct transfer of filtration results from test cells to industrial-sized SWMs seems to be questionable. To overcome the issues of a flat channel test cell, Bu-Ali et al. [38] used five short SWMs of 0.458 m length in series. They observed a pronounced increase in concentration along the flow path, resulting in a longitudinal flux decline and a higher salt concentration in the permeate during RO filtration. As inlet and outlet pressure drops occur in each module, an accurate prediction of filtration performance in SWMs cannot be guaranteed using a series of shorter modules with multiple inlet and outlet flow effects. This can only be achieved by investigating spatial effects in an industrial-sized module.

Even though test cells have some limitations, they are a helpful tool to understand the filtration behavior of polymeric spiral-wound membranes better, or at least to derive filtration trends in full-size SWMs. However, it remains unsolved whether test cells and small-scale applications can predict the filtration performance of SWMs during skim milk MF in a satisfactory manner.

A third option to gain insights into spatial behavior of SWMs are direct and indirect observations. By using nuclear magnetic resonance (NMR) microscopy, Vrouwenvelder et al. [39] showed that the flow distribution in flat channels becomes increasingly inhomogeneous due to fouling. Feed flow was limited to well-defined areas, whereas virtually no feed flow could be observed on the rest of the membrane. Von der Schulenburg et al. [40] applied NMR to characterize flow distribution in SWMs. They observed a heterogeneous distribution of the feed flow in radial direction after biofouling had occurred. Thus, the flow distribution differs in radial and axial direction in a SWM, suggesting that a test cell is not suitable for a precise prediction of the spatial and overall filtration performance of a SWM. An additional measurement is field emission (FE) scanning electron microscopy (SEM), which can be used to determine the deposit layer height in cross-sectioned membranes. Coupling SEM with energy dispersive X-ray (EDX) analysis provides a method to analyze a deposit layer’s composition [41]. Bégoin et al. [41] observed a radial gradient in deposition in cleaned UF membranes after milk filtration using FESEM. Fouling was most pronounced close to the permeate collection tube in the center of the module, confirming an inhomogeneous radial flow distribution. Furthermore, other techniques, such as confocal laser scanning microscopy (CLSM) [42], attenuated total reflection Fourier-transformed infrared spectroscopy (ATR-FTIR) [43], and surface matrix assisted laser desorption ionization mass spectrometry (Surface-MALDI-MS) [44] can be used to analyze the deposit layer. The direct and indirect observation methods, however, cannot provide any insight into spatial flux and protein permeation. 

It can be concluded that the filtration performance of SWMs is subject to spatial effects in the radial and axial directions of the membrane sheets, which, however, has not directly been proven in an industrial-sized SWM to date. Hence, the goal of this study was to develop new in situ test systems for the investigation of spatial effects in SWMs, and to compare them in terms of which best reflected reality. Two different setups of membrane prototypes were constructed: No.1: SWMs of different lengths were used to measure the pressure drop along the flow path. No.2: To analyze flux and protein permeation along a SWM, a standard SWM and the filtration plant were adapted by three modifications: (i) a modification of the membrane itself by dividing the permeate pockets into four compartments to avoid axial mixing; (ii) a modification of the permeate collection tube to collect four different permeate streams; (iii) a modification of the filtration plant to monitor and control spatial effects.

With the membrane prototypes No.1 and No.2, a direct observation of deposit layer formation can only be achieved by destroying the SWM without any chance of reuse. Therefore, a new test cell was constructed and compared to the modified SWM. This needs to be done to assess whether the uncertainty of results obtained by test cells can be eliminated, and whether a more suitable test cell can be developed to overcome the weaknesses and limitations of currently used test cells. With these membrane prototypes and the test cell, the prerequisite to investigate the spatial filtration behavior in SWMs has been created. As with ceramic tubular membranes, the motivation of this study was to create new tools for the generation of data useful for the development of advanced SWMs and/or modes of operation with reduced spatial dependency and a better overall filtration performance.

## 2. Materials and Methods

### 2.1. Preparation of Skim Milk and Calculation of Protein Permeation

Skim milk was used as model fluid to validate the different test systems. Pasteurized skim milk (74 °C, 28 s) was obtained from a local dairy (Molkerei Weihenstephan GmbH & Co. KG, Freising, Germany) and stored at 4 °C for up to four days. The individual casein and whey protein composition of the samples was analyzed by reversed phase high performance liquid chromatography (RP-HPLC) according to Dumpler et al. [45]. Protein permeation (P) was calculated from retentate concentration at 40 min (c_r_, 40 min) and the permeate concentration of a target component (c_p_) by Equation (1).

(1) $$P=\frac{c_{p}}{c_{r,\;40\;\min}}\times 100\%$$

### 2.2. Membranes and Filtration Rigs

The membrane material for all test systems was polyvinylidene fluoride (PVDF) (V0.1, Synder Filtration, Inc., Vacaville, CA, USA) with a nominal pore size of 0.1 µm. The material was either used as flat sheets for the test cell or was manufactured into SWMs by CUT Membrane Technology GmbH (Erkrath, Germany). Prior to the filtration, the membranes were conditioned with caustic Ultrasil 69 (0.4% vol/vol, Ecolab Deutschland GmbH) at 50 °C for 20 min. The cleaning procedure is described in detail elsewhere [46].

#### 2.2.1. Investigation of Spatial Effects on Pilot Scale

**Figure 1:**
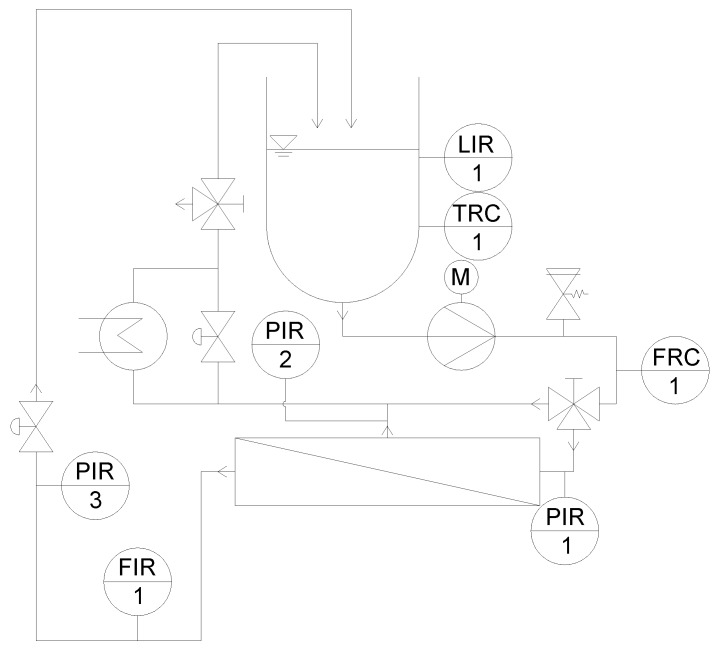
Simplified piping and instrumentation (P&I) diagram of the pilot plant for the investigation on 6338 spiral-wound membranes (SWMs) (modified according to [46]).

Figure 1 shows a simplified piping and instrumentation (P&I) flow chart of the pilot plant used to investigate the SWMs. Details are described elsewhere [46].

In short, this unit consists of a receiver tank (250 L), a multistage centrifugal pump (adjustable from 5 to 20 m^3^ h^−1^ feed volume flow), a heat exchanger for temperature control, and a housing for the SWM. The feed volume flow corresponds to a mean crossflow velocity v between 0.15 and 0.60 m s^−1^ (referring to a 6338 module with a 31 mil spacer). This was calculated by Equation (2), with the feed volume flow $\dot{V}$ and the free cross-section of the membrane without spacer A.

(2) $$v=\frac{\dot{V}}{A}$$

In this configuration, the plant was used to measure the retentate pressure drop in the modules with different lengths. To analyze flux and permeation of proteins along a SWM, a modification of the plant was necessary, which is described in detail in the results section.

#### 2.2.2. Design and Manufacture of Prototype SWM

Prototypes were constructed as 6338 modules with a diameter of 6.3 inches (0.16 m) and a permeate collection tube length of 38 inches (0.96 m). A diamond shaped feed spacer (31 mil) was used and the modules were enveloped in an outer wrap to enhance cleanability. The active membrane length and filtration area were not proportional to the overall length of the modules due to the glue strips in the permeate pockets. Hence, the active membrane area of the sectioned module had to be determined. The total flux of a module is proportional to the active membrane area of a module. Thus, the active membrane area of the sectioned membrane could be calculated by its module specific flux during skim milk filtration at limiting flux conditions, and the corresponding specific flux of the unmodified module at the same conditions. Table 1 summarizes the prototype characteristics.

#### 2.2.3. Validation of SWM Prototypes

Experimental design and start-up procedure are described in detail elsewhere [46]. Filtration experiments were conducted at 10 °C and a native milk pH of 6.8. The SWMs were mounted in a housing (1.3 m) with two cartwheel-shaped anti-telescoping devices. During filtration experiments, retentate and permeate were recirculated and a retentate pressure drop of 0.78 bar m^−1^ was applied (corresponding to a mean crossflow velocity of 0.42 m s^−1^). A Δp_TM_ of 0.5 bar was held for 40 min to ensure steady state filtration was achieved. Subsequently, a gradual increase of Δp_TM_ was carried out in 0.5 bar increments to 3.0 bar, holding each Δp_TM_ level for 30 min. For the Δp_TM_ adjustment, permeate and retentate outlet streams were throttled. Permeate samples were gathered prior to rising the Δp_TM_. Retentate samples were taken in duplicate after 40 min and at the end of the experiment. Flux was measured 20 min after Δp_TM_ adjustment.

#### 2.2.4. Investigation of Spatial Effects on Lab Scale

**Figure 2:**
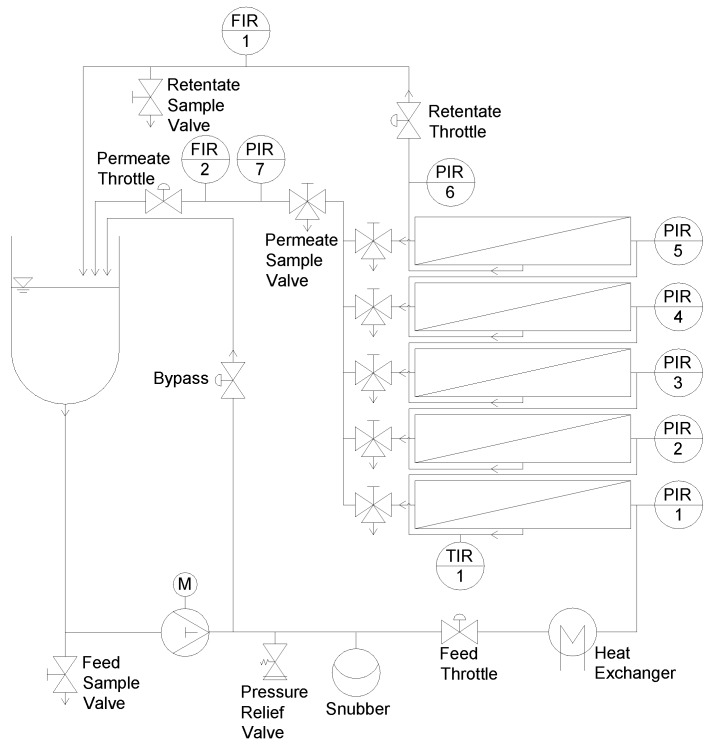
Filtration plant equipped with the filtration unit.

A sectioned flat sheet test cell (SIMA-tec GmbH, Schwalmtal, Germany) was constructed to quantify the deposit layer on membranes. It was equipped with a receiver tank (3 L), a piston pump, a snubber to create a continuous flow, a heat exchanger for feed tempering, and a filtration unit (Figure 2).

**Figure 3:**
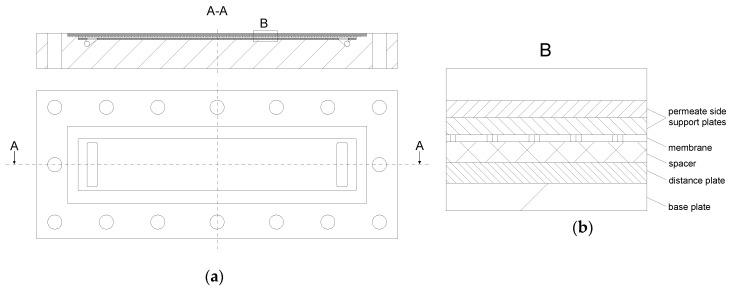
Architecture of a single test cell (**a**). Detail B shows the different inlays in the test cell during a filtration test (**b**).

The filtration unit was built up of five individual test cells (SIMA-tec) connected in series (Figure 3). Therefore, spatial effects could be analyzed at different membrane lengths.

Prior to filtration, a flat sheet membrane piece was mounted in the test cell with the selective layer of the membrane upside down. Before installation, the membrane was soaked in deionized water at room temperature for 24 h. The channel height was designed to allow the placement of spacers up to a height of 114 mil and, thus, was suitable for the investigation of highly viscous feeds. This enhanced the operational area of the test cell compared to other simulators, which have a fixed spacer height [34,35] or a lower range of applicable spacer heights [37]. For spacers with a lower height, distance plates regulated the interspace. The active membrane area was 0.20 m × 0.04 m per test cell. By using five test cells with a length of 0.20 m each, a cumulative membrane length of 1.00 m was achieved, which is similar to the length of one industrial sized SWM. Each of the five test cells was equipped with an outlet to collect individual permeate samples. The retentate pressure before (p_i_) and after (p_i+1_) each test cell plus the permeate pressure (p_p_) was determined using pressure gauges (WIKA Alexander Wiegand SE & Co. KG, Klingenberg am Main, Germany). A mass flow measurement system (Promass 80A01, Endress+Hauser Messtechnik GmbH+Co. KG, Munich, Germany) was used to measure the permeate flux of each section either separately or combined. Therefore, permeates were piped through the mass flow measurement system or through a bypass. Data were logged with an Ecograph T (Endress+Hauser Messtechnik GmbH+Co. KG, Munich, Germany).

#### 2.2.5. Calculation of the Feed Volume Flow in the Test Cell

To create similar hydrodynamic conditions between the test cell and a standard SWM, the feed flow was calculated on basis of a similar mean crossflow velocity by Equation (3). Therefore, the free current cross-section of the SWM and the test cell were put in relation. In Equation (3), the feed volume flow of the test cell ${\dot{V}}_{test\;cell}$, the total membrane area of the SWM *A_SWM_* (22.8 m^2^), the length of the SWM *l_SWM_* (0.96 m), and width of the test cell *b_test cell_* (0.04 m) were used.

(3) $${\dot{V}}_{SWM}=\frac{{\dot{V}}_{test\;cell}\cdot A_{SWM}}{2l_{SWM}\cdot b_{test\;cell}}$$

Referring to a 6338 SWM (length 0.96 m, diameter 0.16 m), hydrodynamic conditions of feed volume flows between 3.0 and 32.7 m³ h^−1^ can be depicted in the test cell by applying feed flows between 10 and 110 L h^−1^. With regard to the 31 mil spacer, this corresponds to a mean crossflow velocity between 0.09 and 0.97 m s^−1^.

#### 2.2.6. Experimental Design

Prior to filtration trials, 2.5 L of skim milk were heated to a process temperature of 10 °C. To remove the mixed phase, the plant was flushed with skim milk. Therefore, 0.5 L were drained before the retentate was recirculated. Following this, the crossflow velocity was adjusted to create a pressure drop of 0.78 bar m^−1^, permeate valves were opened, and Δp_TM_ was adjusted. According to Hartinger et al. [46], Δp_TM_ was gradually increased in steps of 0.5 bar from 0.5 to 3.0 bar. The 0.5 bar Δp_TM_ step was held for 40 min, and the following were held for 30 min to ensure that steady state conditions were reached. The flux was measured 20 min after Δp_TM_ adjustment. Permeate samples were taken prior to rising the Δp_TM_ to the next level and at the end of the experiment. Retentate samples were collected at the end of the 0.5 bar and 3.0 bar steps.

### 2.3. Data Regression and Statistical Analysis

Data were plotted using OriginPro 2017G (OriginLab Corporation, Northampton, MA, USA). Error bars represent the standard deviation of two individual experiments performed with milk from different lots.

## 3. Results and Discussion

### 3.1. Construction of Membranes with Different Lengths

**Figure 4:**
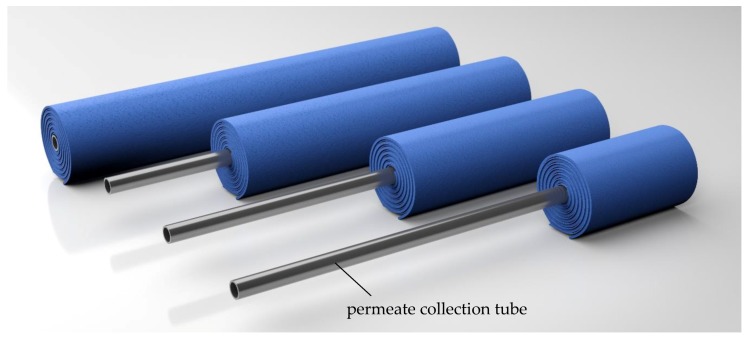
Membranes with different lengths (0.24; 0.48; 0.72; 0.96 m). For all modules, the permeate collection tube has a length of 0.96 m.

The retentate pressure drop directly affects the transmembrane pressure along the membrane, and thus affects the length-dependent deposit layer formation and filtration performance [4]. In order to understand the retentate pressure drop along a complex SWM and compare it to the sectioned module, it is necessary to measure the pressure at different positions along the membrane. In an industrially-sized module (length 0.96 m), it is only possible to measure the pressure at the membrane inlet and outlet. Thus, we constructed SWMs with different membrane lengths (0.24, 0.48, 0.72, 0.96 m) (Figure 4) and installed them in a standard SWM housing. 

In order to achieve flow conditions at the feed inlet similar to a full-length module, the membrane-covered parts of the permeate tube pointed to the feed inlet. The permeate collection tubes had the same length (0.96 m) for each membrane to ensure a correct positioning of the modules in the pressure housing. However, drill holes for the permeate passage into the permeate tube were only present in the active filtration area of the membrane sheets. Apart from that, the membrane prototypes with different lengths were similar to standard SWMs, resulting in an active membrane area of 3.5, 8.6, 13.1, and 19.1 m^2^ for the respective membranes with lengths of 0.24, 0.48, 0.72, and 0.96 m.

### 3.2. Sectioned Membrane Prototype for the Space-Resolved Recording of Flux and Protein Permeation along the Membrane

Aside from the retentate pressure drop, the length-dependent filtration behavior is of particular interest for the evaluation of SWMs. Three independent modifications were necessary to monitor flux and protein permeation along a SWM: a modification of the membrane pockets, the central permeate collection tube, and the filtration plant itself.

#### 3.2.1. Modification of the Membrane Pockets

**Figure 5:**
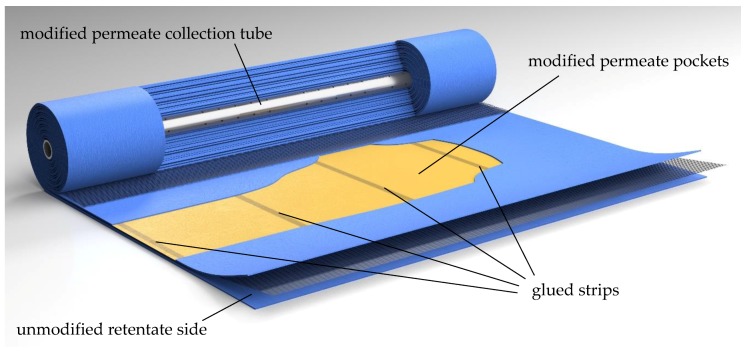
The sectioned module with modified permeate pockets and a modified permeate collection tube. The retentate side is unmodified. For comparison, an unmodified SWM can be found in the supplementary.

A modification of the permeate channel was necessary to avoid axial mixing of permeates originating from different positions in the SWM. Therefore, the permeate pockets were sectioned into four compartments in axial direction prior to winding the membrane sheet around the permeate tube. The sectioning was realized by three equidistant, radially oriented glue strips in each permeate pocket (Figure 5). Thereby, the same technique was applied as for tightening flat sheets to form the permeate pockets. The glue strips reduced the active filtration area of the sectioned SWM by approximately 25% to 14.3 m^2^. However, the glue strips offer another advantage in addition to the avoidance of permeate stream mixing: since no volume can be exchanged between the different compartments, no pressure equalization can take place. Thus, the permeate pressure can be regulated separately in each section. This means that a length-dependent Δp_TM_ adjustment can be carried out by controlling the permeate pressure. Furthermore, the retentate pressure specific to the section can be measured. When the permeate throttles are closed, the pressure in the permeate sections corresponds to the mean pressure on the retentate side, and therefore becomes accessible.

To summarize, the glue strips in the permeate pockets prevent mixing of permeate in axial direction and enable an assessment of the length-dependent filtration performance. By preventing the exchange of liquids between the pockets, permeate pressure and thus Δp_TM_ can be controlled in axial direction in the sectioned SWM.

#### 3.2.2. Modification of the Central Permeate Collection Tube

**Figure 6:**
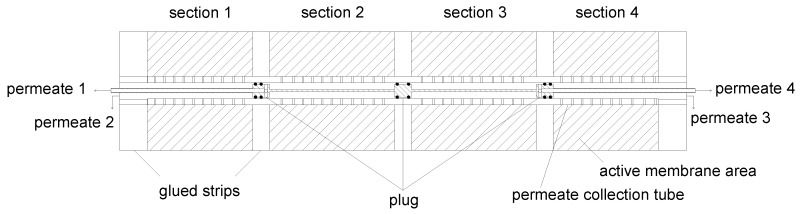
Axial cross-section of a SWM sectioned by glued strips in the permeate pocket, and the plug system integrated into the sectioned SWM. Shaded areas mark the active filtration area.

Since the permeates of the four compartments would mix in the central permeate collection tube, it was additionally necessary to modify the tube. This was implemented by a plug system (Figure 6) with three plugs. Each plug has two O-ring gaskets to divide the collection tube into four compartments sealed against one another. The plugs were positioned at the location of the glue strips. Furthermore, the drill holes in the permeate collection tube were left out in these areas to ensure a homogenous seal face. In order to guarantee a correct positioning of the plugs, they were connected to one another to form a single device.

In this configuration, the plugs would prevent permeate of sections 2 and 3 from draining. Therefore, the outer plugs hold a drill hole in axial direction. Since the permeate of the inner sections (2 and 3) would mix with the permeate of the outer sections (1 and 4), the outer plugs were connected to pipes. This allowss the permeate of the inner sections to flow through the pipes by creating a “tube-in-tube” system. This ensurs that the different permeate streams becomes accessible outside of the SWM. Streams of sections 1 and 2 can be collected on the feed inlet side, streams of sections 3 and 4 on the retentate outlet side.

#### 3.2.3. Modification of the Filtration Plant

**Figure 7:**
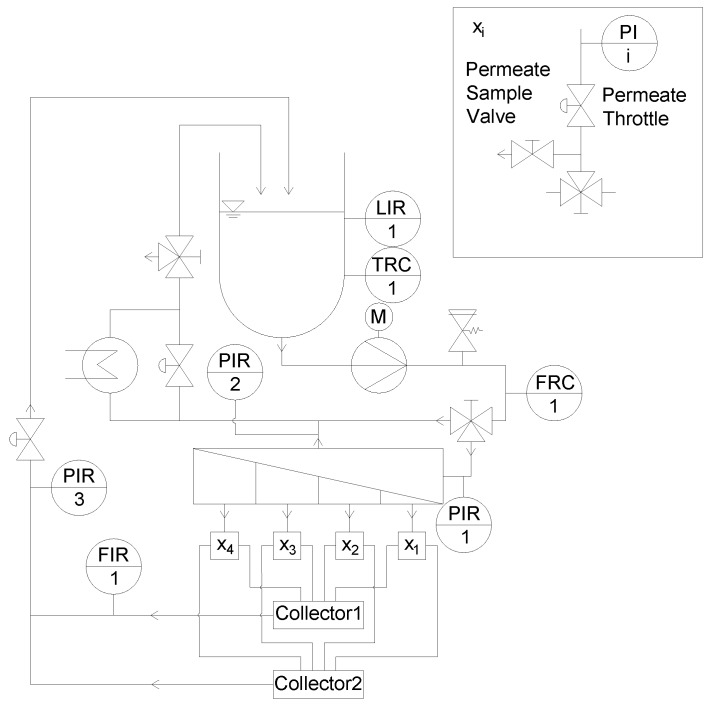
Modified pilot plant, x_1_–x_4_ refer to the set of components and their interconnection, as depicted in the top right box.

In order to be able to analyze, monitor, and control the filtration performance of each section separately, the membrane filtration plant (Figure 1) had to be modified too (Figure 7). For the analysis and quantification of the individual permeate composition of each section, each permeate stream was forced into a separate flow path including a sampler. Four pressure gauges were installed in the four different flow paths to monitor the pressure in each of the four sections. To control the permeate pressure, and thus Δp_TM_, a throttle was integrated in each section. The permeates were directed to either collector 1 or 2 via a three way valve, connected to the flow meter or a bypass. Thus, a selective permeate flow measurement could be performed. After the individual flow measurement, all permeates were merged to a combined permeate stream, which could again be throttled. This design also made it possible to monitor and control the total permeate pressure, and not only the individual sections.

The combined modification of the permeate pockets, the permeate collection tube with the tube-in-tube system, and the filtration plant enables an assessment of length-dependent filtration performance in SWMs.

### 3.3. Validation of the Sectioned Membrane Prototype

**Figure 8:**
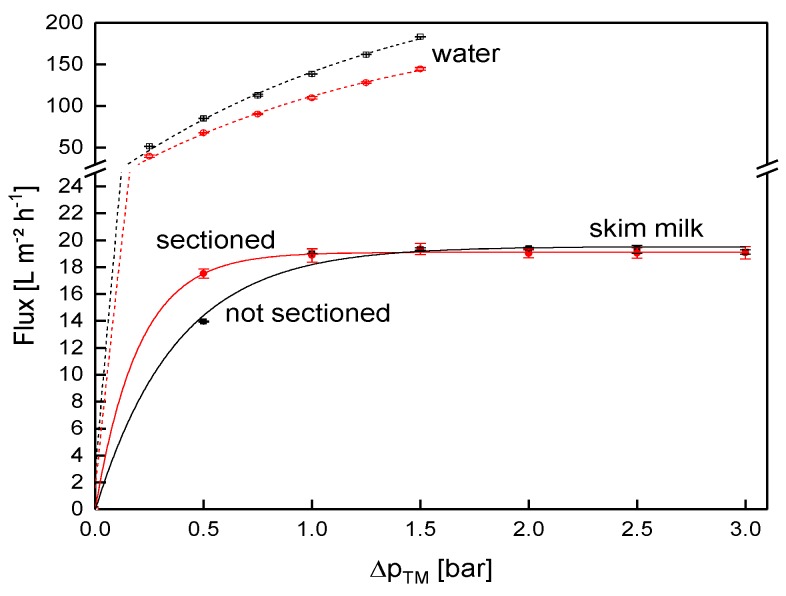
Flux as a function of the transmembrane pressure (Δp_TM_) for the sectioned and a non-sectioned SWM (length of 0.96 m) during filtration of water and skim milk at a mean crossflow velocity of 0.42 m s^−1^.

The next step after the complex set up of the different modifications was to verify that the modifications did not alter the filtration behavior, compared to an unmodified SWM. To monitor the influence of the sectioning, the flux of both SWMs (length of 0.96 m each) as a function of Δp_TM_ was compared (Figure 8). With increasing Δp_TM_, the flux during skim milk filtration increased until the limiting flux of 19 L m^−2^ h^−1^ was reached for both membranes at the same Δp_TM_ of 1.0 bar. Limiting flux conditions were reported in previous studies on skim milk filtration [2,4,46], and were caused by more intense fouling at higher Δp_TM_ values.

Below the limiting flux, there was a slight difference between the two SWMs. In this pressure range (0.5 bar), the filtration performance was rather controlled by the membrane than by the deposit layer, as was the case for the limiting flux. A possible explanation for the deviation is that a pressure alignment appeared in the permeate pocket of the non-sectioned module. More permeate was produced close to the membrane inlet, due to the higher Δp_TM_ [4]. Therefore, permeate flowed to the rear part of the membrane (in axial direction) inside the permeate pocket, and thus increased permeate pressure in the rear of the module. On that account, Δp_TM_ was slightly lower in the rear part compared to the sectioned membrane, where this effect could not occur due to the glued separations. Furthermore, the variability of membrane performance in one batch is known for polymeric membranes. This explains the differences in water flux values between the modified and the unmodified membrane. Overall, however, the deviation was considered acceptable.

**Figure 9:**
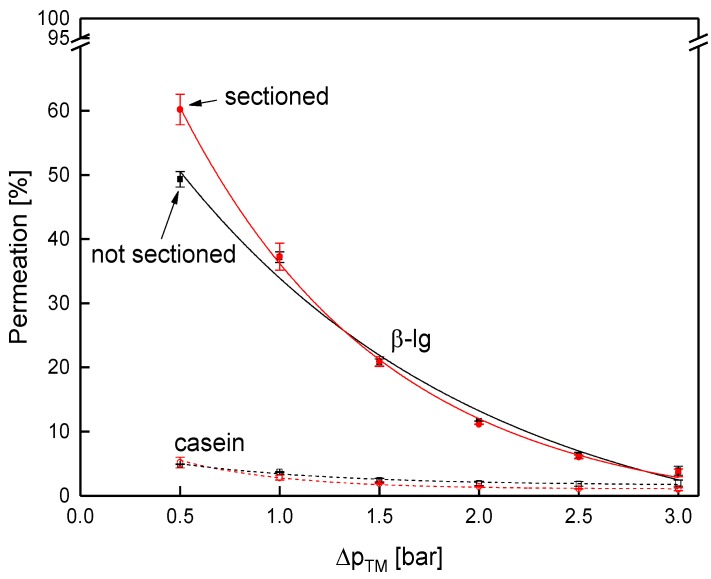
Protein permeation as a function of the Δp_TM_ for the sectioned and the non-sectioned SWM (length of 0.96 m) at a mean crossflow velocity of 0.42 m s^−1^.

Next to the flux, permeation of the major whey protein β-lg as reference for the whey proteins and casein was determined (Figure 9). As known from literature, protein permeation decreases with increasing Δp_TM_ [4,46] and with increasing size of the proteins [10]. Thus, the permeation of both proteins decreased with the Δp_TM_. Further, the casein permeation (size of casein micelles 182 nm [47]) was lower compared to β-lg (4.19 nm [47]), independent of the Δp_TM_. However, accordingly to the flux, the protein permeation was identical for both membranes for both caseins and β-lg. At 0.5 bar, again, a slight difference between the two membranes was observed. The results confirm that filtration performance was practically unaffected by the sectioning.

The conclusion is that both modules give similar results, and thus the sectioned test module can be used to investigate length effects in SWMs.

### 3.4. Spatial Dependency of the Δp_TM_ in SWMs

**Figure 10:**
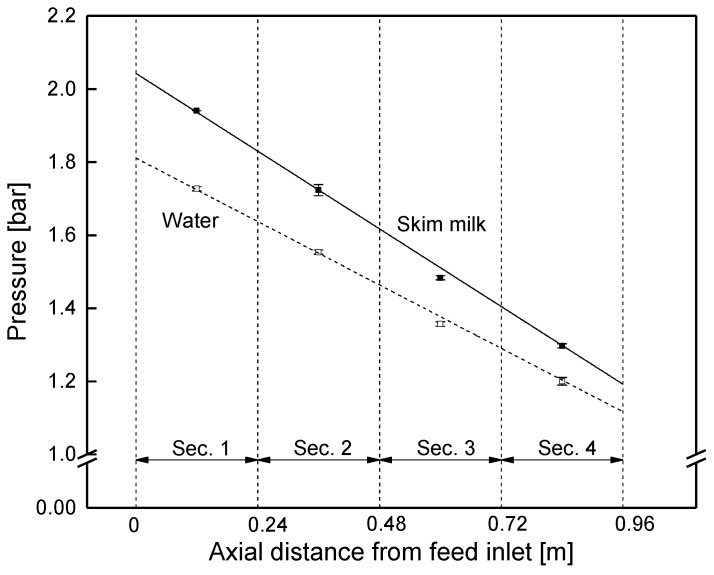
Pressure drop in the sectioned SWM (length of 0.96 m) at a mean crossflow velocity of 0.42 m s^−1^.

Even though it was expected that the retentate pressure would drop linearly along the membrane, this has not yet been proven by data at different positions in an industrial-sized SWM. The pressure drop was studied in the sectioned membrane by closing the permeate valves of each section. Hence, permeate pressure was equal to the average retentate pressure in each section, and thus became measurable via the permeate pressure gauges. Figure 10 shows that the retentate pressure in the module decreased linearly both with water and skim milk. For skim milk, the pressure dropped from an average of 1.94 bar in section 1 to 1.30 bar in section 4. This was due to constant friction in the spacer filled retentate channels [18]. The slope is steeper for milk because its higher viscosity compared to water causes an increase in friction losses.

From the slope of the pressure curve (−0.85 bar m^−1^ for skim milk), the module’s pressure drop was determined to be 0.82 bar at a feed flow of 14 m^3^ h^−1^ (mean crossflow velocity of 0.42 m s^−1^).

**Figure 11:**
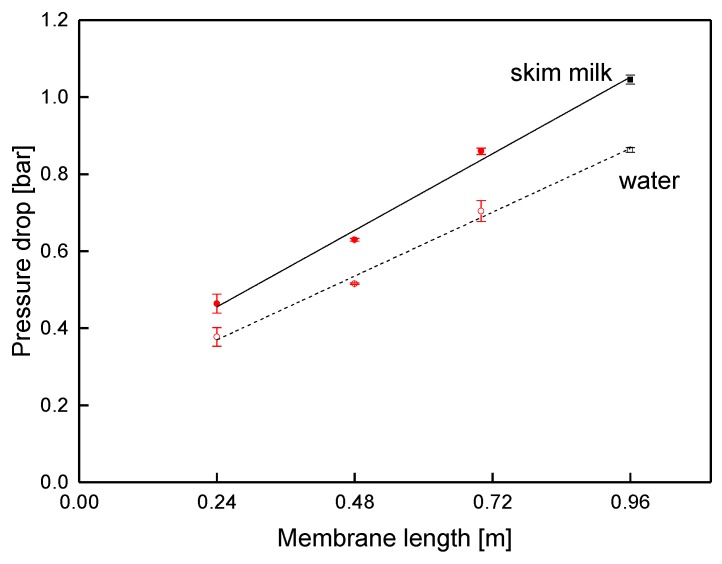
Pressure drop in SWMs of different lengths (red circles: shortened modules; black squares: sectioned membrane) at a mean crossflow velocity of 0.42 m s^−1^.

In order to prove the validity of this approach, the pressure drop was also studied via membranes with different lengths (Figure 11) at a fixed crossflow velocity of 14 m^3^ h^−1^ (mean crossflow velocity of 0.42 m s^−1^). In accordance with the results from the sectioned membrane, the pressure decreased linearly, with a slope of −0.83 and −0.69 bar m^−1^ for skim milk and water, respectively. Since the slope corresponds to the data shown in Figure 10, pressure drop in SWMs can be assessed with both kinds of prototypes, as no effect of the module shape was observable.

At 0.46 bar and 1.04 bar for the SWMs with the length of 0.24 and 0.96 m, respectively, the total pressure drop of skim milk was significantly higher than expected from the slope (0.20 and 0.80 bar). Extrapolated to a membrane length of 0.00 m, the pressure drop was still 0.25 bar. This can be explained as follows: the pressure gauges for the measurement of the overall pressure drop were located outside the membrane. This means that inlet and outlet effects were inevitably included in the measurement at different lengths. The effects are caused by energy dissipation due to a reduction or an enlargement of the flow cross-section, when the feed is forced into or out of the spacer channel. By assessing the slope of the pressure curve, an accurate determination of the pressure drop inside the module is possible.

The results show that the pressure drop inside a SWM is proportional to the length, in accordance with theory. Retentate pressure in SWMs, and thus Δp_TM_, can be approximated with a linear function, as was done with ceramic tubular membranes (Equation (4)) by Piry et al. [4].

(4) $${\Delta p}_{\mathrm{TM},i}=p_{\mathrm{inlet}}-\frac{\left(2\times i-1\right)}{2\times n}\times \left(p_{\mathrm{inlet}}-p_{\mathrm{outlet}}\right)$$

Piry et al. [4] estimated the average Δp_TM_ in each section i by inserting the inlet pressure p_inlet_, outlet pressure p_outlet_, and the total number of sections n. In SWMs, permeate pressure is not negligible, and influences filtration performance [48]. However, the pressure drop in radial direction in the permeate pocket can be assumed to be independent of the axial position. Close to the permeate collection tube, where the highest permeate volume flow is to be expected, the mean crossflow velocity during limiting flux conditions is 0.03 m s^−1^ (calculated by Equation (2)). As flux accumulates in radial direction, the average permeate volume flow in the permeate pocket is lower, resulting in a lower average mean crossflow velocity. Thus, the permeate pressure can be considered to be practically equal in each section, due to the low crossflow velocity.

Considering the permeate pressure and the throttling of the permeate stream, Equation (4) has to be modified by adding the permeate pressure p_p_ (Equation (5)).

(5) $${\Delta p}_{\mathrm{TM},i}=p_{\mathrm{inlet}}-\frac{\left(2\times i-1\right)}{2\times n}\times \left(p_{\mathrm{inlet}}-p_{\mathrm{outlet}}\right)-p_{p}$$

Furthermore, inlet and outlet pressure must be determined according to Equations (6) and (7), using the retentate pressure inside the membrane p_Ret_, the membrane length l, and distance from membrane inlet x.

(6) $$p_{\mathrm{inlet}}=p_{\mathrm{Ret}}\left(x=0\right)$$

(7) $$p_{\mathrm{outlet}}=p_{\mathrm{Ret}}\left(x=l\right)$$

Otherwise, inlet and outlet pressure drops would reduce the accuracy of Equation (5).

As expected, Δp_TM_ decreases linearly in a SWM (compare Equation (5)). By assuming a constant permeate pressure along the module, Δp_TM_ is directly proportional to the retentate pressure. Using Equation (5) allows the assessment of the filtration performance of each section, considering the effective Δp_TM_ and spatial effects.

During filtration with permeate production, the retentate volume flow decreases slightly along the membrane. Since a lower crossflow velocity reduces the pressure drop, it is not completely constant along the membrane, but slightly lower in the rear part of the module. Therefore, the overall pressure drop was insignificantly lower during permeate production, and Equation (5) yields somewhat higher Δp_TM_ values for the rear sections. However, the effect is negligible due to its low extent (max. deviation 0.1 bar m^−1^). Hence, Equation (5) is a suitable tool for the determination of the spatially resolved Δp_TM_ in SWMs.

### 3.5. Comparison of the Test Cell and the Sectioned Membrane Prototype

It has been shown that the introduced prototypes are feasible tools to analyze spatial effects in SWMs. However, SWMs are hardly accessible for investigation and quantification of the deposit layer. For this purpose, a test cell is suitable, as membranes can be removed easily while the deposit layer remains in its position for further analyses.

In the designed test cell, axial disparities in filtration performance can be monitored in five consecutive compartments. Identically to the sectioned SWM, the permeate streams can be collected and controlled individually or collectively by merging the streams of the five sections. To verify whether results of the deposit layer analyses can be related to the observations in a SWM, the two systems were compared to one another.

**Figure 12:**
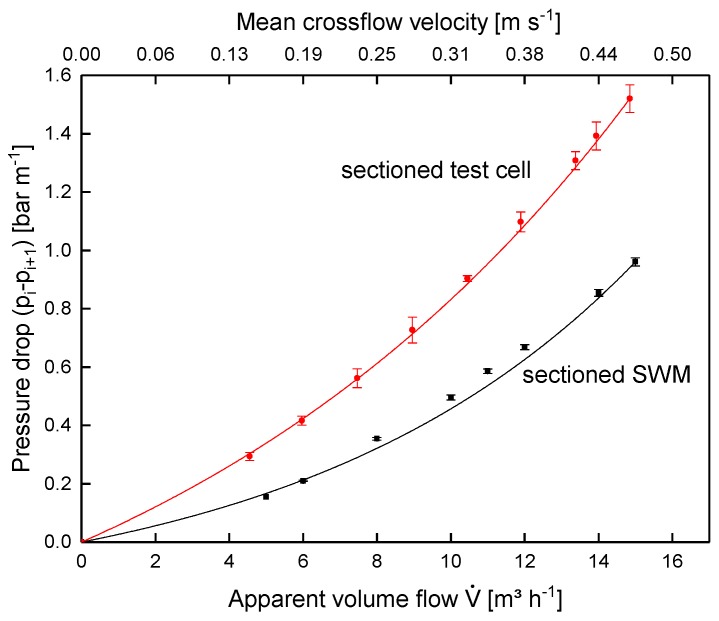
Pressure drop in the sectioned SWM (length of 0.96 m) and the test cell (channel length of 1.00 m) for different volume flows using skim milk.

Figure 12 shows the pressure drop in the sectioned SWM and the test cell. Increasing feed volume flow, and thus crossflow velocity, caused the pressure drop in both systems to increase disproportionately. Furthermore, the absolute pressure drop in the test cell was higher. With an apparent volume flow of 14 m^3^ h^−1^ relating to the SWM (mean crossflow velocity of 0.42 m s^−1^), it was 1.39 and 0.85 bar m^−1^ for the test cell and the sectioned SWM, respectively. This can be explained as follows: in the SWM, a bypass stream through the outer wrap, i.e., between the module housing and the SWM body, reduced the real crossflow velocity and thus pressure drop, although the same apparent feed flow was applied. This is not a weakness of the SWM prototypes, but rather a reflection of reality, as it is also unavoidable in many industrial settings. Siebdrath et al. [37] reported that the pressure drop of their test cell and a SWM matched during water filtration without permeate production. We assume that no bypass occurred in their SWM. Thus, the match is probably not due to the test cell reproducing the flow conditions in the SWM better compared to the test cell in this study, but due to an identical crossflow velocity in the spacer channels.

**Figure 13:**
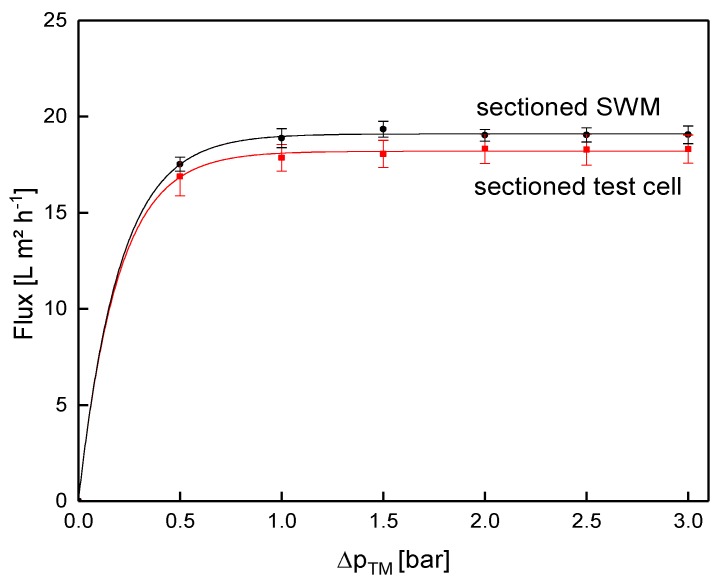
Comparison of flux obtained from the test cell (channel length of 1.00 m) and the sectioned SWM (length of 0.96 m) at a mean crossflow velocity of 0.42 m s^−1^.

**Figure 14:**
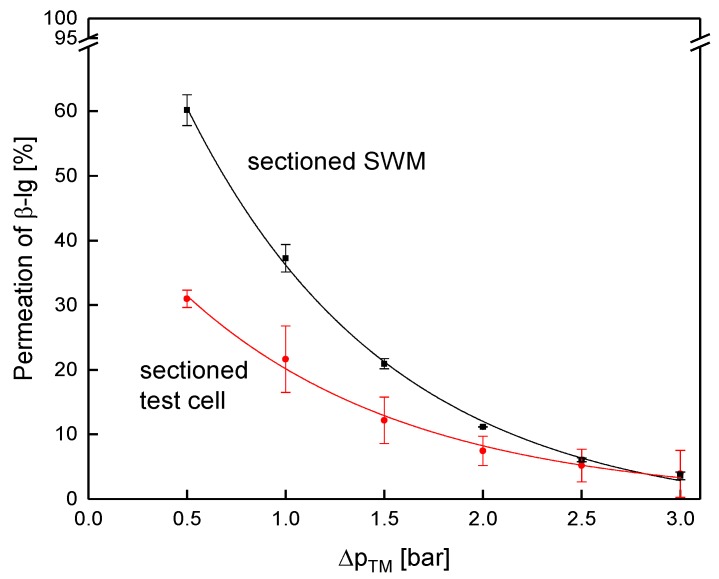
Protein permeation in the test cell (channel length of 1.00 m) and the sectioned membrane (length of 0.96 m) at a mean crossflow velocity of 0.42 m s^−1^.

Since crossflow velocity affects not only the pressure drop, but also deposit layer formation [19], the same pressure drop of 0.78 bar m^−1^ was used for the assessment of flux (Figure 13) and protein permeation (Figure 14). It was assumed that similar flow conditions could be achieved by equalizing the axial pressure drop, and not the feed flow.

In both systems, the flux increased with Δp_TM_ until the limiting flux was reached at about 1.0 bar. The flux of the sectioned test cell (18.3 L m^−2^ h^−1^) was slightly lower compared to the sectioned SWM (19.0 L m^−2^ h^−1^) at limiting flux conditions. In the test cell, small dead zones occurred close to feed inlet and retentate outlet. Therefore, the crossflow velocity was not fully developed, except for the area between inlet and outlet. As deposit layer formation was much more pronounced in the boundary areas, the flux was therefore reduced. Although these areas contribute to the active membrane surface, the cumulated flux in the test cell was slightly reduced. However, this influence can be neglected due to its small extent, as flux performance of the sectioned test cell and the sectioned SWM were practically the same.

Contrary to that, protein permeation was considerably lower in the sectioned test cell compared to the sectioned membrane (Figure 14). At 0.5 bar, permeation of β-lg was 32% and 60%, respectively.

As for flux, dead zones close to feed inlet and retentate outlet, as well as boundary effects, reduced filtration performance. Due to different in channel geometries and boundary effects, the test cell cannot reproduce exact values for filtration performance of SWM. The absolute values of the pressure drop between both systems differ, due to a bypass current through the outer space of the SWM between housing and membrane body.

Considering this, filtration tests were executed at an equal pressure drop, and not at the same apparent crossflow velocity. It was expected that in this case, the effective crossflow velocity in the spacer filled channels and the flow pattern would be practically equal for both systems. Regarding filtration performance, the flux was similar at an equal pressure drop, whereas protein permeation was lower for the sectioned test cell, due to boundary effects. Consequently, the test cell is not capable of depicting exact performance values, but filtration trends are similar in both systems. Therefore, spatial effects in SWMs and the influence of process variables on filtration performance of SWMs can be monitored in the test cell. The advantage of the designed test cell compared to SWMs is that membranes can be assessed immediately after filtration mitigating alterations in the deposit layer structure. To our knowledge, no data exist on the time needed to extract membranes from existing flat sheet test cells. The extraction process of the membrane from the test cell in this study took less than 5 min for the first membrane sheet, and about 1 min for each of the following 4 membrane sheets. Thus, changes in the deposit layer due to diffusion are reduced to a minimum.

To conclude, the test cell system can provide additional information on the filtration process, since it allows fast and non-destructive access to the membranes for further analyses, such as on the deposit layer formation.

## 4. Conclusions

The main goal of this study was to develop and validate new concepts and membrane prototypes suitable for analyzing, monitoring, and controlling spatial effects in SWMs. Shortened SWMs were constructed with the purpose of measuring the pressure drop in SWMs. A second approach was to construct a sectioned membrane to analyze spatial dependency of flux and protein permeation along an industrial-sized SWM. The sectioning was realized by three modifications: sectioning of the membrane pockets by glued strips on the permeate side, sectioning of the permeate collection tube, and modifying the filtration plant. It was shown that sectioning a membrane does not practically affect filtration performance compared to an unmodified SWM. Thus, the developed SWM can be used for the spatially resolved investigation of the filtration performance, which has not been possible with existing industrial SWMs.

For ceramic membranes, length-dependent filtration performance is directly related to the Δp_TM_. To assess spatial filtration performance in SWMs, retentate pressure drop was investigated with both SWM prototype systems, proving that it decreases linearly in axial direction. Since the permeate pressure is practically constant in each section, the spatially resolved Δp_TM_ can be determined in SWMs, similarly to ceramic membranes. This facilitates the evaluation of filtration performance in relation to the Δp_TM_. To conclude, the designed membrane prototypes are valuable tools to further analyze and understand spatial effects in SWMs, as the filtration performance can be assessed in situ.

However, one limitation of the prototype SWM is that direct access to the membrane sheets after filtration is not possible without destruction of the modules. Thus, the deposit layer cannot be analyzed directly. Therefore, a new test cell system was designed. This test cell allows access to the membrane immediately after filtration. The system, thus, seems to be suitable for the analysis of deposit layer formation relative to SWMs in areas with fully developed crossflow conditions. This has to be investigated in a further study.

Apart from that, the test cell system cannot accurately predict filtration performance of SWMs, confirming the need for the SWM prototypes for the assessment of spatial effects. As similar filtration trends occur in the sectioned test cell and the SWM, the test cell can provide additional information on the influence of process variables on the deposit layer formation during MF with polymeric membranes. To conclude, the combination of the developed membrane prototypes and the test cell represents a well-suited toolbox to understand the spatial dependency in SWMs.

## Figures and Tables

**Table 1:** Characteristics of prototype membranes.

Membrane Specification	Length [m]	Active Filtration Area [m²]
Membranes with different lengths	0.24	3.5
	0.48	8.6
	0.72	13.1
	0.96	19.1
Sectioned membrane	0.96	14.3

## Supplementary Materials

The following are available online at https://www.mdpi.com/2077-0375/9/7/80/s1.
