# Supplementary figures and images for: Technical Concepts for the Investigation of Spatial Effects in Spiral-Wound Microfiltration Membranes
Source: Membranes (Basel). 2019 Jul 4;9(7):80. doi: 10.3390/membranes9070080 (PMC6680769; doi:10.3390/membranes9070080)

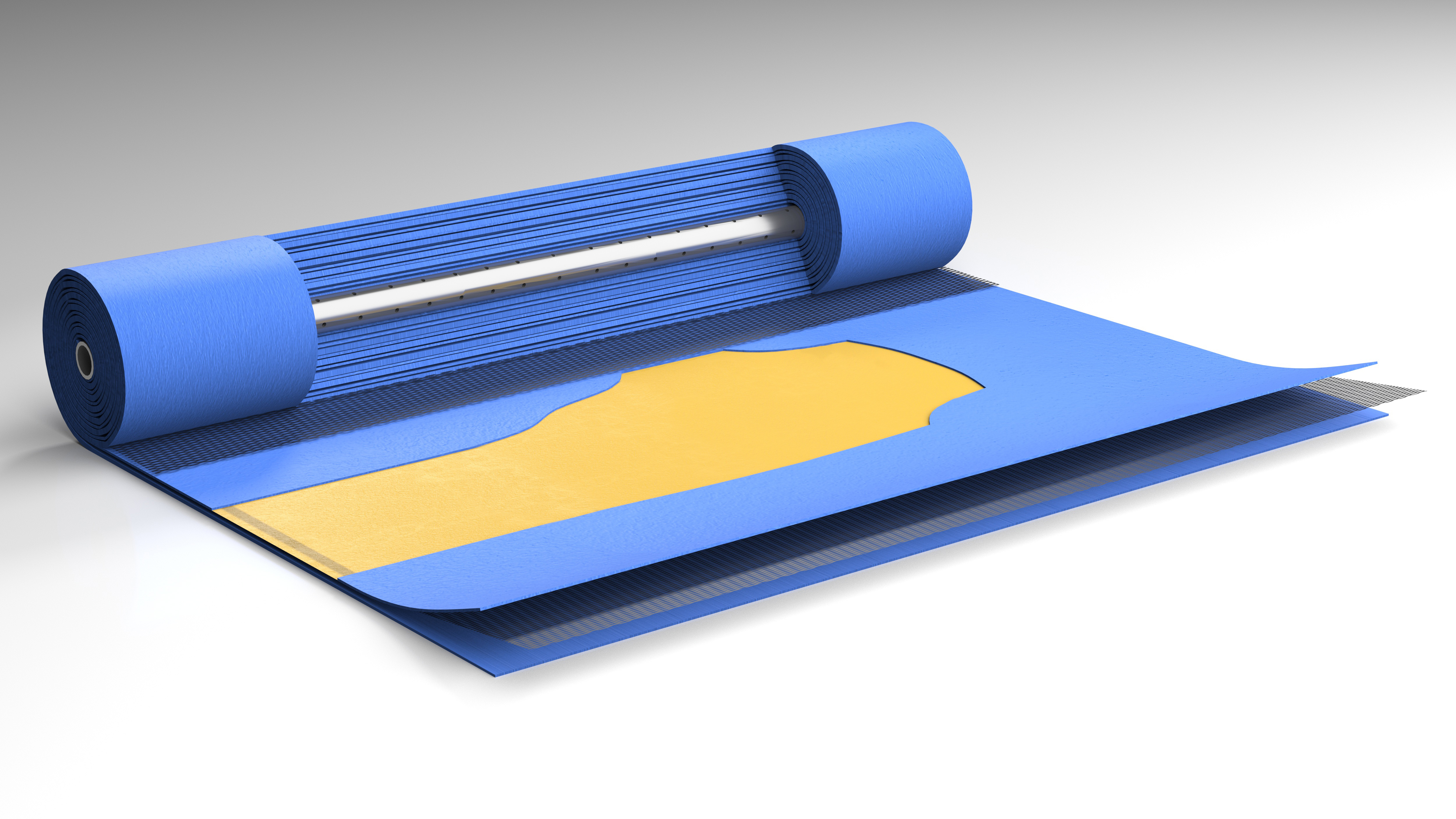

Supplement: Supplementary file 1 [file membranes-09-00080-s001.zip › membranes-526035-supplementary.jpg]
